# News and Notes

**Published:** 1902-11

**Authors:** 


### NEWS AND NOTES.

(We are indebted to the *Medical Age* for many of these notes.)

---

THE marriage of Dr. H. F. Hansell, U. S. A., and Miss Annie Wilson occurred on October 24.

---

DR. CHAS. H. FRAZIER has been appointed dean of the University of Pennsylvania Medical School.

---

THE Mississippi Medical Association held its annual meeting at Kansas City on October 15, 16, and 17.

---

PROFESSOR LANNELONGUE, of Paris, has presented the Paris Académie de Médecine with $7,600 for the endowment of a triennial prize.

---

YELLOW FEVER is reported to be epidemic in Orizaba, a city on the line of the Mexican railroad, between Vera Cruz and Mexico City.

---

W. H. WELCH, professor of pathology in the Johns Hopkins University, recently sailed for England to deliver the Huxley lectures at the Charing Cross Hospital.

---

IT is announced that Colonel Charles Smart, who has been ordered to the Philippines as chief surgeon of the division, will make a thorough investigation of the origin of the cholera bacillus with a view to provide for the adoption of permanent measures to check the recurring epidemics of cholera in the islands.

IN a recent U. S. P. H. and M. H. report, Dr. J. O. Cobb, who investigated the disease called spotted fever, prevailing in the Bitter Root Valley, Mont., found that the disease was caused by the bite of a tick. These ticks are parasites upon the ground squirrel, and the spread of the disease has been traced to the section of the country infested with ground squirrels.

---

SURGEON OLIVER D. MORTON, U. S. Navy, says the *Journal of the American Medical Association*, has been authorized to accept a present tendered him by the Emperor of Germany. Former recipients of honors were Assistant-Surgeon William J. Wilson, U. S. Army, a decoration from the Khedive of Egypt, for gallantry in battle; Surgeon-General Rufus Tryon, U. S. Navy, a medal from the government of Venezuela; and the late medical inspector, George Wood, U. S. Navy, a decoration from the Queen of Hawaii.

---

THE Fellows of the Georgia Pasteur Institute held their second annual meeting in Atlanta October 3d. President Slack's report showed that the past had been a very successful year. Forty-three cases had been successfully treated, and no case has died after treatment has been completed. The Board of Governors has purchased a nice brick house at 85 Luckie street, which will be the permanent home of the Institute. The following Board of Governors and Officers were elected: Henry R. Slack, M.D., President, LaGrange; J. H. McDuffie, M.D., Vice-President, Columbus; C. D. Hurt, M.D., Treasurer, Atlanta; Jas. N. Brawner, M.D., Sec. and Physician, Atlanta; Claude A. Smith, M.D., Pathologist, Atlanta; Benj. W. Hunt, Eatonton; E. P. Ham, M.D., Gainesville; George C. Walters, Atlanta; M. M. Holland, M.D., Statesboro.

